# Matrix Metalloproteinase-9 (MMP-9) induced disruption of intestinal epithelial tight junction barrier is mediated by NF-κB activation

**DOI:** 10.1371/journal.pone.0249544

**Published:** 2021-04-07

**Authors:** Rana Al-Sadi, Jessica Engers, Mohammad Haque, Steven King, Deemah Al-Omari, Thomas Y. Ma

**Affiliations:** 1 Department of Medicine, Penn State University College of Medicine, Milton S. Hershey Medical Center, Hershey, Pennsylvania, United States of America; 2 Department of Biology, University of New Mexico, Albuquerque, New Mexico, United States of America; Emory University School of Medicine, UNITED STATES

## Abstract

**Background:**

Matrix Metalloproteinase-9 (MMP-9) has been shown to play a key role in mediating inflammation and tissue damage in inflammatory bowel disease (IBD). In patients with IBD, the intestinal tight junction (TJ) barrier is compromised as characterized by an increase in intestinal permeability. MMP-9 is elevated in intestinal tissue, serum and stool of patients with IBD. Previous studies from our laboratory showed that MMP-9 causes an increase in intestinal epithelial TJ permeability and that the MMP-9 induced increase in intestinal permeability is an important pathogenic factor contributing to the development of intestinal inflammation in IBD. However, the intracellular mechanisms that mediate the MMP-9 modulation of intestinal barrier function remain unclear.

**Aims:**

The main aim of this study was to further elucidate the molecular mechanisms involved in MMP-9 induced increase in intestinal epithelial TJ permeability using Caco-2 monolayers as an *in-vitro* model system.

**Results:**

MMP-9 induced increase in Caco-2 TJ permeability was associated with activation and cytoplasmic-to-nuclear translocation of NF-κB p65. Knocking-down NF-κB p65 by siRNA transfection prevented the MMP-9 induced expression of the NF-κB target gene IL-8, myosin light chain kinase (MLCK) protein expression, and subsequently prevented the increase in Caco-2 TJ permeability. In addition, the effect of MMP-9 on Caco-2 intestinal epithelial TJ barrier function was not mediated by apoptosis or necrosis.

**Conclusion:**

Our data show that the MMP-9 induced disruption of Caco-2 intestinal epithelial TJ barrier function is regulated by NF-κB pathway activation of MLCK.

## Introduction

The intestinal epithelium is a single layer of cells within the gut lumen that serves an important protective function. It acts as a selectively permeable barrier, which allows for absorption of nutrients while preventing harmful luminal contents, such as gut microbes, from crossing the intestinal epithelium [1, 2]. Defective intestinal tight junction barrier (TJ), manifested by an increased intestinal permeability, serves as a key contributor to the pathogenesis of inflammatory bowel disease (IBD) including Crohn’s disease (CD) and ulcerative colitis (UC), celiac disease, and other inflammatory conditions of the gut [3, 4]. Clinical studies in IBD patients have shown the key role of intestinal TJ barrier function in the pathogenesis of intestinal inflammation and emphasized on the enhancement of intestinal barrier which correlated with sustained long-term clinical remission [5, 6].

Recently, matrix metalloproteinases (MMPs) have been implicated in the pathogenesis of intestinal inflammation in IBD [7–9]. MMPs are classified into major subgroups including collagenases (MMP-1, -8, -13, -18), gelatinases (MMP -2, -9), stromelysins (MMP -3, -7, -10, -11, -19), elastase (MMP -12), and membrane type (MMP -1, -5) [8, 10]. Under normal physiologic conditions, MMPs are involved in processes including angiogenesis, degradation and remodeling of extracellular matrix (ECM), wound repair, and the activation of a wide range of inflammatory cytokines [11, 12]. Dysregulated MMP expression, however, can lead to an exaggerated and prolonged inflammatory response, leading to chronic inflammation [12, 13]. MMP-9 has been postulated to be proinflammatory and its levels are shown to be markedly elevated in intestinal tissue, serum, and stool of patients with IBD and closely correlate with the disease activity [14, 15]. Previous studies have shown that in animal models of colitis, MMP-9 was upregulated and played an important role in the development of intestinal inflammation [16–19]. The dextran sodium sulfate (DSS) and the trinitrobenzene sulfonic acid (TNBS)-induced colitis were inhibited in MMP deficient mice [17, 20, 21]. Furthermore, it has been shown that MMP-9 inhibition (by a pharmacologic inhibitor or by genetic knock down) prevented the intestinal inflammation in an ileus model of enterocolitis [22]. Together, all previous data suggested the critical role of MMP-9 in mediating the development of intestinal inflammation. Little is known about the mechanisms of MMP-9 induced increase in intestinal TJ permeability, and even less has been reported on whether MMP-9 has a critical role in the pathogenesis of IBD in relation to the TJ barrier function. Our recent studies suggested that MMP-9 at clinically relevant concentrations causes an increase in intestinal epithelial TJ permeability *in vitro* and *in vivo* [23]; however, the intracellular mechanisms involved in MMP-9 induced increase in intestinal TJ permeability remain unclear. The purpose of this study was to further elucidate the intracellular mechanisms involved in the MMP-9 induced increase in intestinal epithelial TJ permeability, using a well-established *in vitro* intestinal epithelial model system consisting of filter-grown Caco-2 monolayers. Our results showed that the MMP-9 induced increase in Caco-2 TJ permeability was mediated by an activation of NF-κB signaling pathway. Identification of intracellular and molecular processes involved in the MMP-9 disruption of intestinal TJ barrier function could be important in developing new restorative approaches to enhance the intestinal TJ barrier and subsequently in preventing intestinal inflammation.

## Materials and methods

### Reagents

DMEM, trypsin, FBS, glutamine, penicillin, streptomycin, PBS and horseradish peroxidase (HRP)-conjugated secondary antibodies for Western blot analysis were purchased from Invitrogen Life Technologies (San Francisco, CA). MLCK antibody was obtained from Sigma (St. Louis, MO). Phospho-NF-κB p65 and IκB-α, NF-κB p65 and β-actin antibodies were purchased from Santa Cruz (Dallas, TX). SiRNA of NF-κB p65 (RelA), and transfection reagents were obtained from Dharmacon (Lafayette, CO). The active form of MMP-9 was purchased from Abcam (Cambridge, MA), isolated from stimulated human neutrophil granulocytes, disulfide-bridged MMP-9 homodimer. All other chemicals were of reagent grade and were purchased from Sigma-Aldrich (St. Louis, MO), VWR (Aurora, CO), or Fisher Scientific (Pittsburgh, PA).

### Cell culture

Caco-2 cells (passage 20) were purchased from the American Type Culture Collection-ATCC (Manassas, VA) and maintained at 37°C in a culture medium composed of DMEM with 4.5 mg/ml glucose, 50 U/ml penicillin, 50 U/ml streptomycin, 4 mM glutamine, 25 mM HEPES, and 10% FBS. The cells were kept at 37°C in a 5% CO_2_ environment. For growth on filters, high-density Caco-2 cells (1 X 10^5^ cells) were plated on Transwell filters with 0.4-μm pore (Corning, Corning, NY) and monitored regularly by visualization with an inverted microscope and by epithelial resistance measurements. Caco-2 monolayers were cultured for 3–4 weeks after seeding and only Caco-2 cells from passages 21 to 28 were used to maintain consistency. The filter-grown Caco-2 monolayers have been used extensively over the last 20 years as *in vitro* model system of functional epithelial barrier [24].

### Determination of epithelial monolayer resistance and paracellular permeability

The transepithelial electrical resistance (TER) of the filter-grown Caco-2 intestinal monolayers was measured using an epithelial voltohmeter (EVOM; World Precision Instruments, Sarasota, FL) as previously reported [23, 25]. Electrical resistance was measured in 5% difference on three consecutive measurements. Caco-2 paracellular permeability was assessed by measuring the luminal-to-serosal flux rate of a paracellular probe, fluorescein isothiocyanate-labeled FITC dextran 10 kDa (mol wt.: 10,000 g/mol). For determination of mucosal-to-serosal flux rates, known concentration (25 μg/ml) of FITC dextran 10 kDa was added to the apical solution at the beginning of each experiment. After each experimental period, the solution from the basolateral chamber was collected and measured in a fluorescence microplate reader (Biotek Flx 800). All flux studies were carried out at 37°C and filter-grown Caco-2-monolayers having epithelial resistance of 400–550 Ω·cm^2^ were used. All of the permeability experiments were repeated three to four times in triplicate.

### Assessment of protein expression by Western blot analysis

Protein expression from Caco-2 cells was assessed by Western blot analysis, as previously described [23, 26]. Cells were lysed with lysis buffer (50 mM Tris·HCl, pH 7.5, 150 mM NaCl, 500 M NaF, 2 mM EDTA, 100 M vanadate, 100 M PMSF, 1 g/ml leupeptin, 1 g/ml pepstatin A, 40 mM paranitrophenyl phosphate, 1 g/ml aprotinin, and 1% Triton X-100) on ice for 30 min. The lysates were centrifuged at 1000 *g* for 10 min in an Eppendorf centrifuge (5417R; Hauppauge, NY) to obtain a clear lysate. The supernatant was collected and protein concentration was determined using the Bio-Rad Protein Assay kit (Bio-Rad, Hercules, CA). Laemmli gel loading buffer (Bio-Rad) was added to the lysate containing 10–20 μg of protein and boiled at 100°C for 7 min, after which proteins were separated on an SDS-PAGE gel. Proteins from the gel were transferred to a nitrocellulose membrane overnight. The membrane was incubated for 2 hrs in blocking solution (5% dry milk in TBS-Tween-20 buffer) and then incubated with antibody in blocking solution. After a wash in TBS-1% Tween buffer, the membrane was incubated in secondary antibody and developed using enhanced chemi-luminescence reagents on ChemiDoc Gel Imaging (Biorad, Hercules, Ca).

### Transfection of siRNA NF-κB p65

Targeted siRNAs were obtained from Dharmacon (Chicago, IL). Caco-2 monolayers were transiently transfected using DharmaFect transfection reagent. Briefly, 5 x 10^5^ cells per filter were seeded into a 12-well transwell plate and grown to confluence. Caco-2 monolayers were then washed twice with PBS and 0.5 ml of Accell Media was added to the apical compartment of each filter and 1.5 ml was added to the basolateral compartment of each filter. Five nanograms of the siRNA and 2 μl of DharmaFect reagent were added to the apical media. Non-target (NT) siRNA was used as a control. The MMP-9 experiments were carried out 72 hrs after transfection. The efficiency of silencing was confirmed by Western blot analysis.

### Nuclear extracts and ELISA

Filter-grown Caco-2 cells were treated with MMP-9 for 60 minutes. Nuclear and cytoplasmic fractions were extracted according to the manufacturer’s protocol using Nuclear Extract Kit from ActiveMotif [27]. The NF-κB DNA-binding activity assay was performed using Trans-AM ELISA-based kits from Active Motif according to the manufacturer’s protocol. In brief, the binding reactions contained 1 pM biotinylated probe (Integrated DNA Technologies) and 5 μg of nuclear extract in complete binding buffer with a total volume of 50 μl. After 30 min of incubation, the solution was transferred to an individual well on the plate and incubated for 1 h. Rabbit NF-κB p65 Ab (2 μg/ml) was added to the well to bind NF-κB p65 from the nuclear extract. After incubation for 1 hr, NF-κB p65 Ab was removed, and 100 μl of anti-rabbit HRP-conjugated IgG were added to the well and incubated for 1 hr. Subsequently, 100 μl of developing solution was added for 2–10 min, and 100 μl of stop solution were added. The absorbance at 450 nm was determined using the SpectraMax 190 (Molecular Devices).

### Immunostaining of NF-κB p65

Cellular localization of the transcription factor NF-κB p65 was assessed by an immunofluorescent Ab-labeling technique as previously described [28]. At the end of the experimental period, filter-grown Caco-2 monolayers were washed twice in cold PBS and were fixed with methanol for 20 min. Then, cells were permeabilized with 0.1% Triton X-100 in PBS at room temperature for 20 min. The Caco-2 monolayers were then incubated in blocking solution composed of BSA and normal donkey serum in PBS for 1 hr. Cells were then labeled with primary Abs in blocking solution overnight at 4°C. After being washed with PBS, the filters were incubated in FITC-conjugated secondary Ab for 1 hr at room temperature. Prolong Gold antifade reagent with DAPI was used to mount the filters onto the coverslips. Immunostaining of NF-κB p65 was visualized and images obtained using a Nikon fluorescence microscope equipped with Axiocam digital camera in automatic mode. Images were processed with Zen software (Zeiss).

### Measurement of IL-8

Caco-2 monolayers were treated with MMP-9 (400 ng/ml) for 24 hrs in serum-free DMEM medium. The culture media was then collected and centrifuged and IL-8 levels were then measured using Multi-Analyte ELISArray kit from Qiagen according to the manufacturer’s protocol.

### Assessment of apoptosis and cell death

After MMP-9 experimental treatment (24 and 48 hrs), Caco-2 cells were trypsinized and assessed for apoptosis using the Annexin V^FITC^ Apoptosis Detection kit II from Biolegend according to the manufacturer’s instructions. Cells were digested by 0.25% trypsin-EDTA solution, washed twice with cold PBS and stained with Annexin V-PE (5 μL) and 7-amino-actinomycin D (7-AAD) (5 μL) in binding buffer. Annexin V^FITC^ was used to stain for the apoptotic cells and 7-AAD was used to stain the necrotic cells. Cells were washed two times with FACS staining buffer and analyzed by 16-color BD LSR Fortessa. Treated or untreated cells were washed and 20,000 cells were re-suspended in 20ul of trypan blue. Cells were counted under a microscope by using a cell counting hemocytometer. Trypan blue-stained dead or necrotic cells were excluded from the total number of cells.

### Statistical analysis

Results are expressed as mean ± SD of triplicate measurements. Two-way ANOVA were performed to determine whether different treatments affect the outcomes. Multiple comparisons were conducted following significant ANOVA by using *t*-tests. All analyses were performed using GraphPad Prism (GraphPad Prism 7.00 for Windows, GraphPad Software). A *P* value of 0.05 was used to indicate statistical significance. All experiments were repeated at least three times to ensure reproducibility.

## Results

### NF-κB requirement in MMP-9 induced increase in Caco-2 TJ permeability

**Fig 1:**
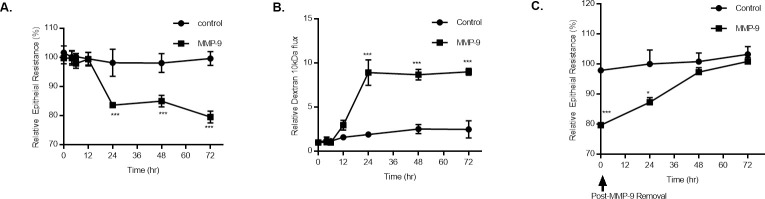
Time-course effect of MMP-9 on Caco-2 intestinal epithelial TJ permeability. (A) Time-course effect of MMP-9 (400 ng/ml) on Caco-2 TER and (B) mucosal-to-serosal flux of paracellular marker dextran 10 kDa (n = 4). *** *P* < 0.001 vs control. The effect of MMP-9 on Caco-2 TER and paracellular permeability were measured over a 72-hr experimental period. (C) Effect of recovery after MMP-9 treatment cessation on Caco-2 TER (n = 4). *** *P* < 0.001 vs control; * *P* < 0.05 vs control. Filter-grown Caco-2 monolayers were refreshed with control media after MMP-9 experimental period ended.

In these experiments, MMP-9 (400 ng/ml) caused a time-dependent drop in Caco-2 trans-epithelial resistance (TER) (Fig 1A), as also shown previously by us [23], MMP-9 caused time-dependent drop in Caco-2 TER (25%) between 24 and 72 hrs of treatment. Conversely, MMP-9 caused a correlated increase in Caco-2 permeability to a paracellular marker, dextran 10 kDa (Fig 1B) starting at 12 hrs of MMP-9 treatment and continued up to 72 hrs of treatment (~ 10-fold). The time course of Caco-2 TER recovery following MMP-9 removal was also examined. The removal of MMP-9 after 72 hrs of treatment resulted in recovery of the Caco-2 TER starting at 24 hrs and continued up to 72 hrs of removal until full recovery was achieved (Fig 1C).

**Fig 2:**
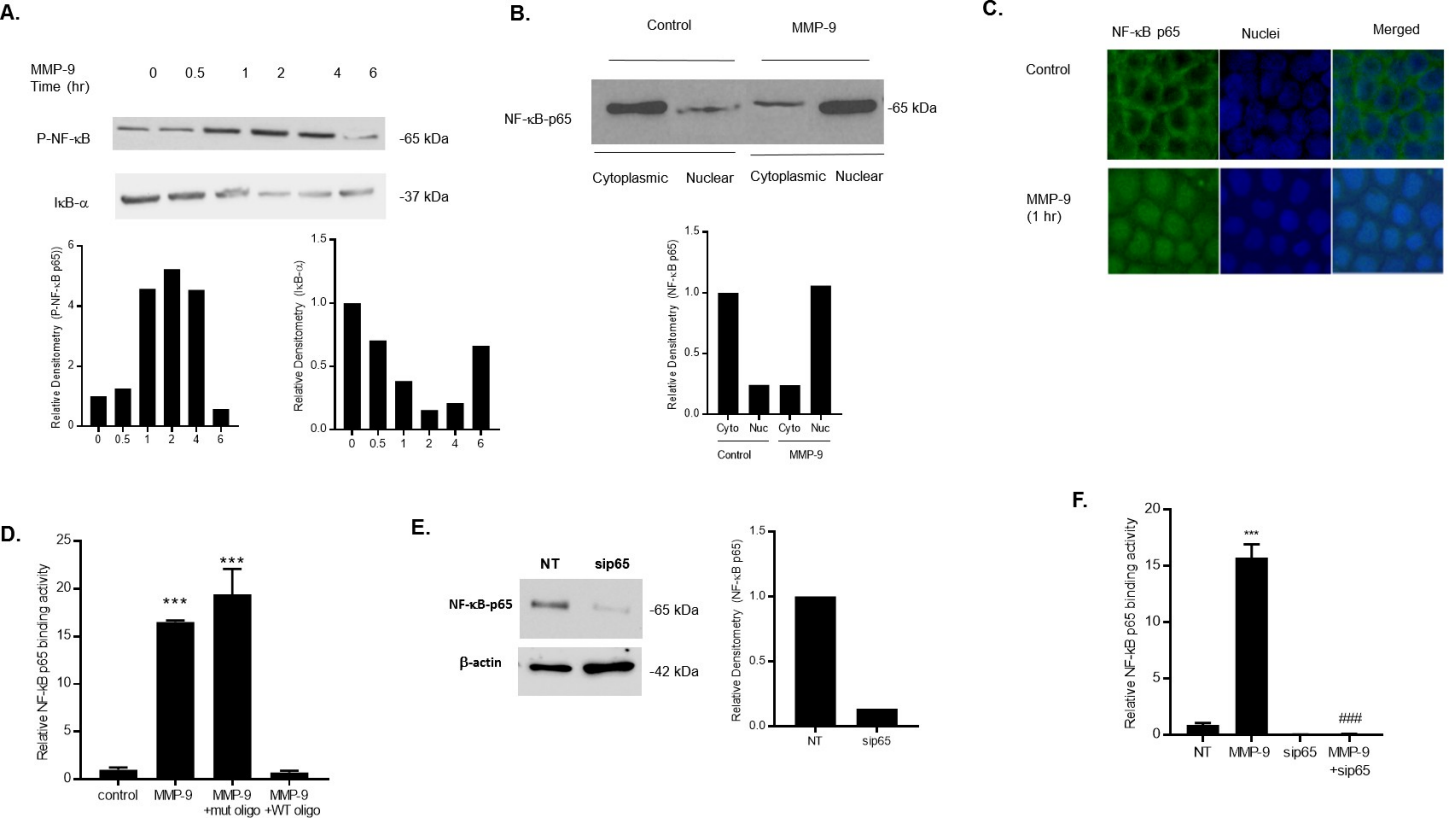
Effect of MMP-9 on NF-κB p65 activation in Caco-2 monolayers. (A) Phospho-NF-κB p65 and IκB-α expression were determined in filter-grown Caco-2 monolayers treated with MMP-9 (400 ng/ml) for increasing time periods (0–6 hrs). (B) NF-κB p65 expression in the cytoplasmic and nuclear fractions was assayed by Western blot analysis after MMP-9 treatment (1-hr experimental period). (C) NF-κB p65 cytoplasmic-to-nuclear translocation was determined by immunostaining. (Yellow, NF-kB p65; Blue, DAPI (nuclei)). Original magnification, ×40. (D) NF-κB p65 binding to the oligonucleotide probe containing the κB-binding site was determined by ELISA-binding assay. MMP-9 caused a significant increase in NF-κB p65 binding (1-hr experimental period). The oligonucleotide containing a mutated NF-κB-binding (mut) motif did not inhibit the NF-κB p65 binding to the DNA probe; however, the addition of wild-type (WT) oligonucleotide containing the consensus NF-κB p65-binding site as a competitive inhibitor prevented the binding of NF-κB. *** *P* < 0.001 vs control. (E) NF-κB p65 siRNA transfection resulted in a marked depletion in NF-κB p65 protein expression. Caco-2 monolayers were transfected with NF-κB p65 siRNA for a 72-hr time period, (NT; not-target siRNA). (F) NF-κB p65 siRNA knock-down prevented the MMP-9 induced increase in NF-κB p65 binding to the oligonucleotide probe containing the κB-binding site. *** *P* < 0.0001 vs control; ^###^ *P* < 0.001 vs MMP-9 treatment.

Many studies have reported the important role of NF-κB activation in regulating the intestinal TJ barrier function *in vitro* and *in vivo* [29–36]. In these experiments, we examined the possible involvement of NF-κB in MMP-9 induced increase in Caco-2 TJ permeability. MMP-9 treatment caused a time-dependent increase in phosphorylated NF-κB p65, peaking at 1 hr of treatment and continued up to 4 hrs of treatment (Fig 2A). Since IκB-α degradation is required for the activation of NF-κB p65, we also examined the effect of MMP-9 on IκB-α degradation in Caco-2 monolayers. MMP-9 caused a time-dependent degradation of IκB-α, indicating that MMP-9 caused activation of NF-κB p65 (Fig 2A).

To further validate the MMP-9 activation of NF-κB p65 in Caco-2 monolayers, we examined the effect of MMP-9 on cytoplasmic-to-nuclear translocation of NF-κB p65 by measuring NF-κB p65 expression in the cytoplasmic and nuclear fractions. In control Caco-2 monolayers, NF-κB p65 is expressed mostly in the cytoplasmic fraction, with only minimal amount in the nuclear fraction (Fig 2B). MMP-9 treatment resulted in cytoplasmic NF-κB p65 translocation into the nuclear fraction (Fig 2B). MMP-9 induced NF-κB p65 cytoplasmic-to-nuclear translocation was also examined by immunostaining. MMP-9 treatment resulted in a rapid increase in NF-kB p65 cytoplasmic-to-nuclear translocation following MMP-9 treatment (1-hr experimental period) (Fig 2C), confirming that MMP-9 causes activation and translocation of NF-κB p65 in Caco-2 monolayers. Next, we examined the effect of MMP-9 on NF-κB p65 activity by assessing the nuclear binding of NF-κB p65 to its consensus sequence by ELISA-binding assay. As shown in Fig 2D, MMP-9 caused a significant and marked increase in NF-κB p65 binding to the consensus κB sequence (5′-GGGACTTTCC-3′) on the oligonucleotide probe, suggesting an increase in NF-κB p65 transcriptional activity in response to MMP-9 in Caco-2 monolayers. The specificity of NF-κB p65 binding to the DNA probe was confirmed by adding a high dose of wild-type oligonucleotide containing the consensus κB-binding site as a competitive inhibitor for NF-κB p65 binding. As expected, the addition of wild-type oligonucleotide inhibited the binding of NF-κB p65 to the DNA probe. In contrast, the mutated NF-κB-binding motif (5′-CTCACTTTCC-3′) did not inhibit the NF-κB p65 binding (Fig 2D), indicatingthat MMP-9-activated and -transclocated NF-κB p65 binds to a κB site on DNA. To further investigate the requirement of NF-κB p65 in the MMP-9 induced increase in Caco-2 intestinal TJ permeability, we knocked-down the expression of NF-κB p65 by siRNA transfection in filter-grown Caco-2 monolayers. As shown in Fig 2E, knocking-down NF-κB p65 by siRNA transfection resulted in a near-complete silencing of p65 expression, and completely inhibited the MMP-9 induced increase in NF-κB p65 nuclear binding to its DNA probe (Fig 2F).

### NF-κB p65 requirement for MMP-9 induced increase in MLCK expression

**Fig 3:**
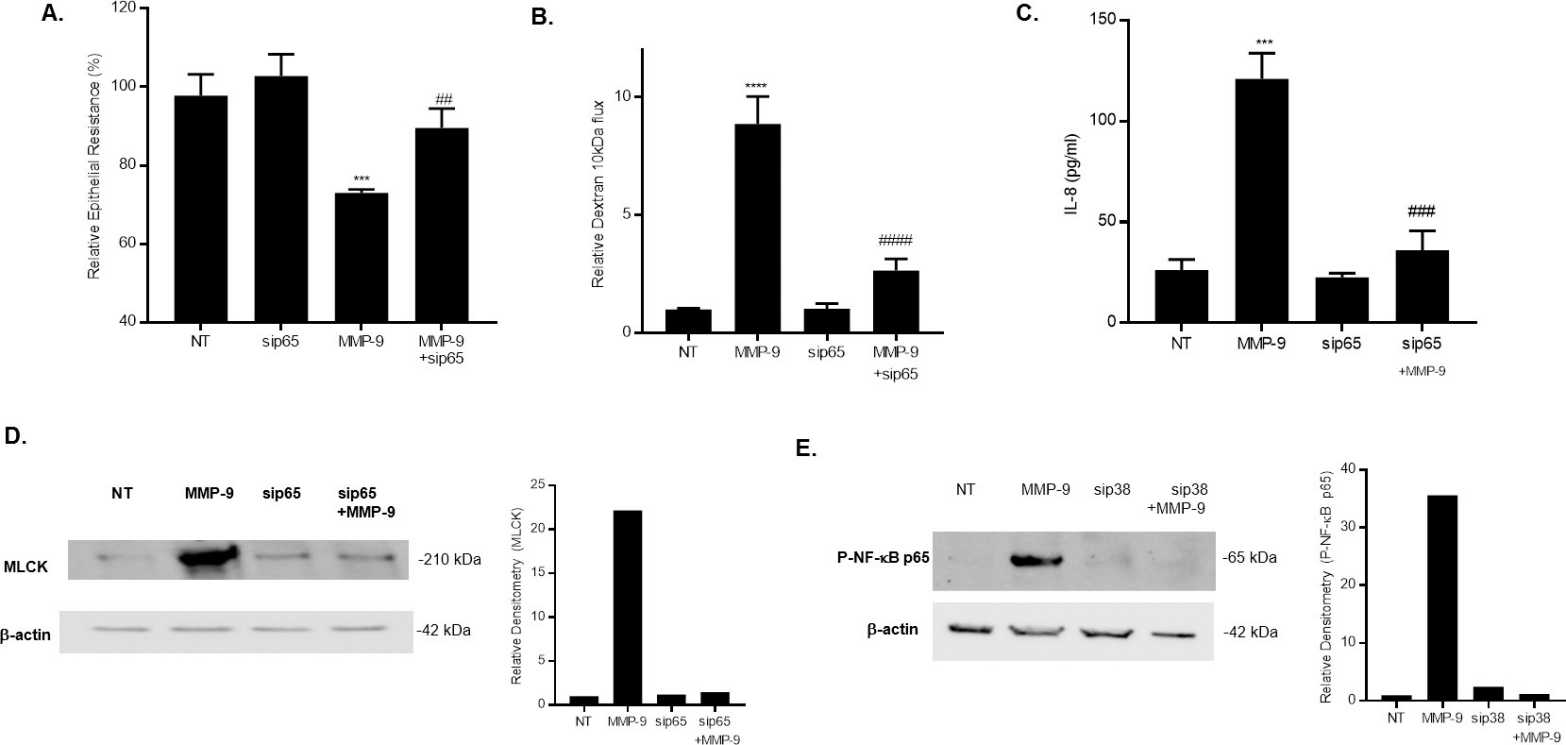
Effect of NF-κB inhibition on MMP-9 induced increase in Caco-2 TJ permeability and increase in MLCK expression. (A) NF-κB p65 siRNA transfection prevented the MMP-9-induced drop in Caco-2 TER and (B) increase in dextran 10 kDa flux (n = 4). *** *P* < 0.001 vs control; ^##^ *P* < 0.01 vs MMP-9 treatment; **** *P* < 0.0001 vs control; ^####^ *P* < 0.0001 vs MMP-9 treatment. (C) MMP-9 treatment resulted in a significant increase in NF-κB target gene IL-8. NF-κB p65 siRNA prevented the IL-8 production by Caco-2 monolayers after MMP-9 treatment (24 hrs). IL-8 secretion was determined by collecting and centrifuging the media and then assayed by ELISA-based kit. *** *P* < 0.0001 vs control; ^###^ *P* < 0.001 vs. MMP-9 treatment. (D) Knocking-down NF-κB p65 inhibited the MMP-9 induced increase in MLCK protein expression as assessed by western blot analysis. (NT; non-target siRNA). (E) Silencing p38 kinase by siRNA transfection prevented the MMP-9-induced activation of NF-κB p65 as assessed by phosphorylation of p65. Caco-2 monolayers were transfected with p38 kinase siRNA for a 72-hr time period and then treated with MMP-9 for 1 hr, (NT; not-target siRNA).

In the following studies, we showed that knocking-down NF-κB p65 by siRNA transfection prevented the MMP-9 induced drop in Caco-2 TER and increase in dextran 10 kDa flux (Fig 3A and 3B), suggesting a crucial regulatory role of NF-κB p65 in the MMP-9 induced increase in Caco-2 intestinal TJ permeability. Furthermore, we examined the effect of MMP-9 treatment on NF-kB down-stream target gene IL-8 production in Caco-2 monolayers. Fig 3C showed that MMP-9 treatment resulted in a significant increase in IL-8 levels in medium collected from Caco-2 cultures as measured by ELISA. Knocking-down NF-kB p65 by siRNA prevented the MMP_9 induced increase in IL-8 production, suggesting that MMP-9 activation of NF-kB is associated with an increase in the release of the pro-inflammatory cytokine IL-8.

Recent studies from our laboratory indicated that the MMP-9‐induced increase in intestinal TJ permeability *in vitro* and *in vivo* was mediated by an increase in MLCK protein expression and activity [23]. However, the intracellular pathways involved in the MMP-9 regulation of MLCK gene and protein expression remain unclear. We examined the possibility that the NF-κB p65 pathway regulates the MMP-9 induced up‐regulation of MLCK protein expression. MMP-9 caused an increase in Caco‐2 MLCK protein expression (Fig 3D), and knocking-down NF-κB p65 by siRNA transfection inhibited the MMP-9 induced increase in MLCK expression (Fig 3D). These findings indicated that MMP-9 activation of the NF-κB p65 pathway mediated the increase in MLCK expression. It has also been reported that MMP-9 modulation of MLCK expression was mediated by p38 kinase activation [23]. To examine the role of p38 kinase in MMP-9-activation of NF-κB in Caco-2 monolayers, we silenced p38 kinase by siRNA transfection and determined MMP-9 activation of NF-κB p65 in Caco-2 monolayers. SiRNA p38 kinase prevented the MMP-9 induced activation/phosphorylation of NF-κB p65 (Fig 3E), suggesting that NF-κB p65 regulation of MLCK is down-stream of p38 kinase

### MMP-9 induced increase in Caco-2 TJ permeability is not due to apoptosis

**Fig 4:**
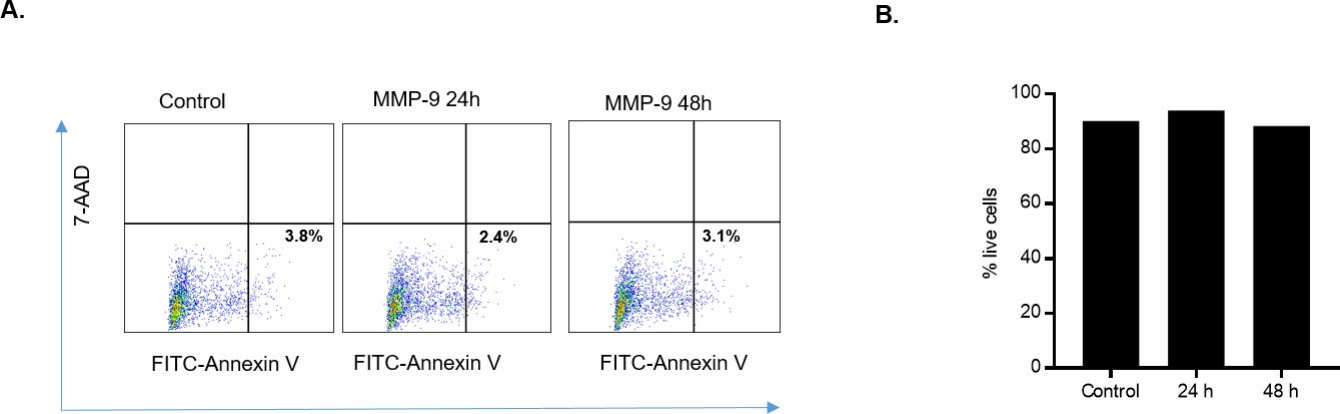
Effect of MMP-9 on Caco-2 cell death. (A) Dual-color dot plots representing cellular apoptosis. Upper left panel, Necrotic cells; lower right panel, apoptotic cells. Caco-2 monolayers were treated with MMP-9 for 24 and 48 hrs. Caco-2 monolayers were labeled with Annexin V^FITC^ (apoptosis) or 7-AAD (necrosis). MMP-9 did not induce apoptosis or cell necrosis in Caco-2 monolayers. (B) % of live cell detection by trypan blue exclusion method. MMP-9 treatment did not affect total number of dead Caco-2 cells compared to control.

MMP-9 has been previously shown to play controversial pro-apoptotic and anti-apoptotic roles in different cell types [37–45]. In the following studies, we examined whether MMP-9 induced disruption of Caco-2 TJ barrier was due to apoptosis or cell death. MMP-9 effect on Caco-2 cell apoptosis and necrosis was determined by flow cytometry by labeling the apoptotic cells with Annexin V^FITC^ and necrotic cells with 7-amino-actinomycin D (7-AAD) [46]. Apoptosis quantification was determined by detecting membrane phosphatidylserine using Annexin V as a probe. While, necrosis was determined by measuring the membrane integrity where 7-AAD is able to permeate across the cell membrane to label the DNA. As shown in Fig 4, MMP-9 treatment did not result in Caco-2 cell apoptosis or necrosis, indicating that the MMP-9 induced increase in Caco-2 TJ permeability was due to intracellular mechanisms not involving apoptosis or cell death.

## Discussion

Despite its central importance in mediating the inflammatory process in IBD and animal models of colitis, the intracellular mechanisms of MMP-9 regulation of intestinal TJ barrier function have not been identified. The present study demonstrated the involvement of NF-κB p65 in MMP-9 induced increase in intestinal epithelial TJ permeability. The results showed that MMP-9 at a physiologically relevant concentration (400 ng/ml) caused rapid and early activation of NF-κB p65 in filter-grown Caco-2 monolayers. Moreover, knocking-down NF-κB p65 by siRNA prevented the MMP-9 induced increase in Caco-2 TJ permeability. Additionally, silencing NF-κB p65 prevented the MMP-9 induced up-regulation of MLCK, a key regulator of the intestinal TJ barrier. Moreover, the MMP-9 induced increase in Caco-2 intestinal epithelial TJ permeability was not mediated by apoptosis.

MMP-9 is one of the metalloproteinases family consisting of 25 members [47]. MMP-9 is unique as its protein expression and activity is undetectable in most healthy intestinal tissues but has been shown to be highly expressed in a variety of inflammatory states, including IBD [48]. It has been shown recently that MMP-9 caused an increase in intestinal epithelial TJ permeability in Caco-2 monolayers [23]; however, the intracellular mechanisms involved have yet to be delineated. In this study, we examined potential intracellular mechanisms that mediate the MMP-9 induced increase in Caco-2 intestinal epithelial TJ barrier. Extensive studies have indicated that the transcription factor NF-κB plays a crucial role in regulating the intestinal TJ barrier function and increased permeability *in vitro* and in animal models of colitis [30, 33, 49–53], and inhibition of NF-κB signaling improved symptoms of DSS-induced colitis in mice [54, 55]. It is also worth noting that NF-κB activity was found to be increased in patients with IBD [56]. Moreover, previous studies have provided compelling evidence on the crucial role that the NF-κB signaling pathway plays in mediating the cytokine-induced increase in intestinal epithelial TJ permeability, including TNF-α, IL-1β, IL-6, IL-8, IFN-γ, IL-13 and others [26, 30, 57–62]. Although there appears to be an existing relationship between NF-κB activity and downstream activation of MMP-9 in intestinal inflammation, there is no clear evidence of MMP-9 activating NF-κB to modulate the intestinal epithelial TJ barrier. For example, it has been shown that TNF- α induced the expression of MMP-9 in human bronchial epithelial cells and that this induction is mediated via the NF-κB -mediated pathway [63, 64]. Earlier studies have shown that *Helicobacter pylori* induced activation of NF-κB, leading to MMP-9 gene transcription in gastric epithelial cells [65]. More recent studies have shown that aluminum caused an increase in HT-29 intestinal epithelial TJ permeability that was mediated by NF-κB induced up-regulation of MMP-9 [66]. In addition to their roles in increasing intestinal permeability, the current study showed the sequential relationship of MMP-9 activating the downstream effector NF-κB to regulate the intestinal epithelial TJ barrier function. MMP-9 caused a rapid degradation of IκB-α and phosphorylation of NF-κB p65 subunit in Caco-2 cells. The requirement of NF-κB p65 in the MMP-9 induced increase in TJ permeability was demonstrated by the silencing of NF-κB p65 expression in Caco-2 cells. NF-κB p65 depletion by NF-κB p65 siRNA transfection significantly attenuated the MMP-9-induced increase in Caco-2 TJ permeability. In combination, these studies confirmed the requirement of NF-κB in mediating the MMP-9 induced increase in Caco-2 TJ permeability. Interestingly, our results showed that inhibition of NF-κB also prevented the MMP-9 activation of the NF-κB target gene IL-8. This finding is consistent with previous reports demonstrating the requirement of intestinal epithelial MMP-9 in mediating intestinal inflammation through increase secretion of IL-8 and disruption of intestinal epithelial barrier in a genetically engineered mouse model that can overexpress MMP-9 specifically in intestinal epithelial cells [67]. Additional investigation is needed to delineate the MMP-9 induced mechanisms *in vivo*, including possible recruitment of immune cells that produce pro-inflammatory cytokines leading to further disruption of the intestinal epithelial TJ barrier.

MMP-9 had been proposed to trigger apoptosis in many cell types [68–70] and the increase in apoptosis in intestinal epithelial cells has been postulated to be an important mechanism for the leakiness observed in IBD patients [71–73]. Due to these findings, we examined the possibility that the MMP-9 induced increase in Caco-2 TJ permeability may also be due to an increase in cell apoptosis. MMP-9 treatment over different time periods did not induce apoptosis or necrosis in Caco-2 cells, suggesting that Caco-2 cell apoptosis or cell death was not a mechanism mediating the MMP-9-induced increase in Caco-2 TJ permeability. The role of MMP-9 in apoptosis has been controversial: previous studies have shown that MMP-9 up-regulation had a protective effect in colitis associated cancer (CAC) and induced apoptosis via activation of Notch-1 signaling [37, 39]; other studies have shown that knock-out models of MMP-9 resulted in delayed apoptosis of hypertrophic chondrocytes [74]. It is worth noting that apoptosis has been suggested to be an important intracellular mechanism for some cytokine (TNF-α, IFN-γ IL-4, and IL-13)-induced increase in intestinal TJ permeability [51, 75–77]. In line with our current studies, NF-κB has been shown to protect cells from death by inducing expression of anti-apoptotic proteins, including Bcl-x_L_, FLICE-like inhibitory protein, and members of the inhibitor of apoptosis (IAP) family [78, 79]. In contrast, other reports have shown that NF-κB activation in epithelial cells caused an increase in the production of inflammatory chemokines that recruit immune cells to tissues, thereby initiating an inflammatory amplification cascade and acting in a pro-apoptotic manner [80]. Since we have found herein that MMP-9 caused an increase in intestinal epithelial TJ permeability in an apoptosis-independent manner, future studies identifying the anti-apoptotic proteins involved will be of great insight into understanding the intracellular mechanisms required in MMP-9 induced increase in intestinal TJ permeability and barrier defect.

Previous studies from our laboratory and others have shown the central role of myosin light chain kinase (MLCK) in regulating the intestinal epithelial TJ permeability [81–84]. MLCK is a Ca^2+^-calmodulin-dependent serine/threonine kinase that has been shown to regulate peri-junctional acto-myosin filaments and mechanical induced opening of the TJ barrier in intestinal epithelial cells. Moreover, previous studies have found that increased MLCK expression activity strongly correlated with active inflammation in IBD [85]. MLCK has also been shown to be essential in mediating the TNF-α, IL-1β and IL-13-induced increase in intestinal epithelial TJ permeability *in vitro* and *in vivo* [25, 30, 34, 85–88]. In addition, several studies have found a pathogenic role of MLCK in both intestinal barrier dysfunction and intestinal inflammation in animal models of IBD [88–90]. Recently, we showed that MMP-9 induced increase in intestinal epithelial TJ permeability was also mediated by an increase in MLCK expression in a p38 kinase-dependent manner *in vitro* and *in vivo* and in a DSS-colitis mouse model [23, 91]. However, the involvement of NF-κB in MMP-9 induced increase in MLCK expression was not determined. In the current study, we demonstrated the requirement of NF-κB activation in MMP-9-upregulation of MLCK expression in Caco-2 monolayers. Silencing of the NF-κB p65 subunit prevented the MMP-9 induced increase in MLCK expression and subsequent increase in intestinal epithelial TJ permeability. Consistent with the current data, previous studies have shown that the NF-κB signaling pathway mediated the cytokine-induced MLCK activation leading to an MLCK-dependent increase in intestinal TJ permeability *in vitro* and *in vivo* [30, 88, 92, 93].

**Fig 5:**
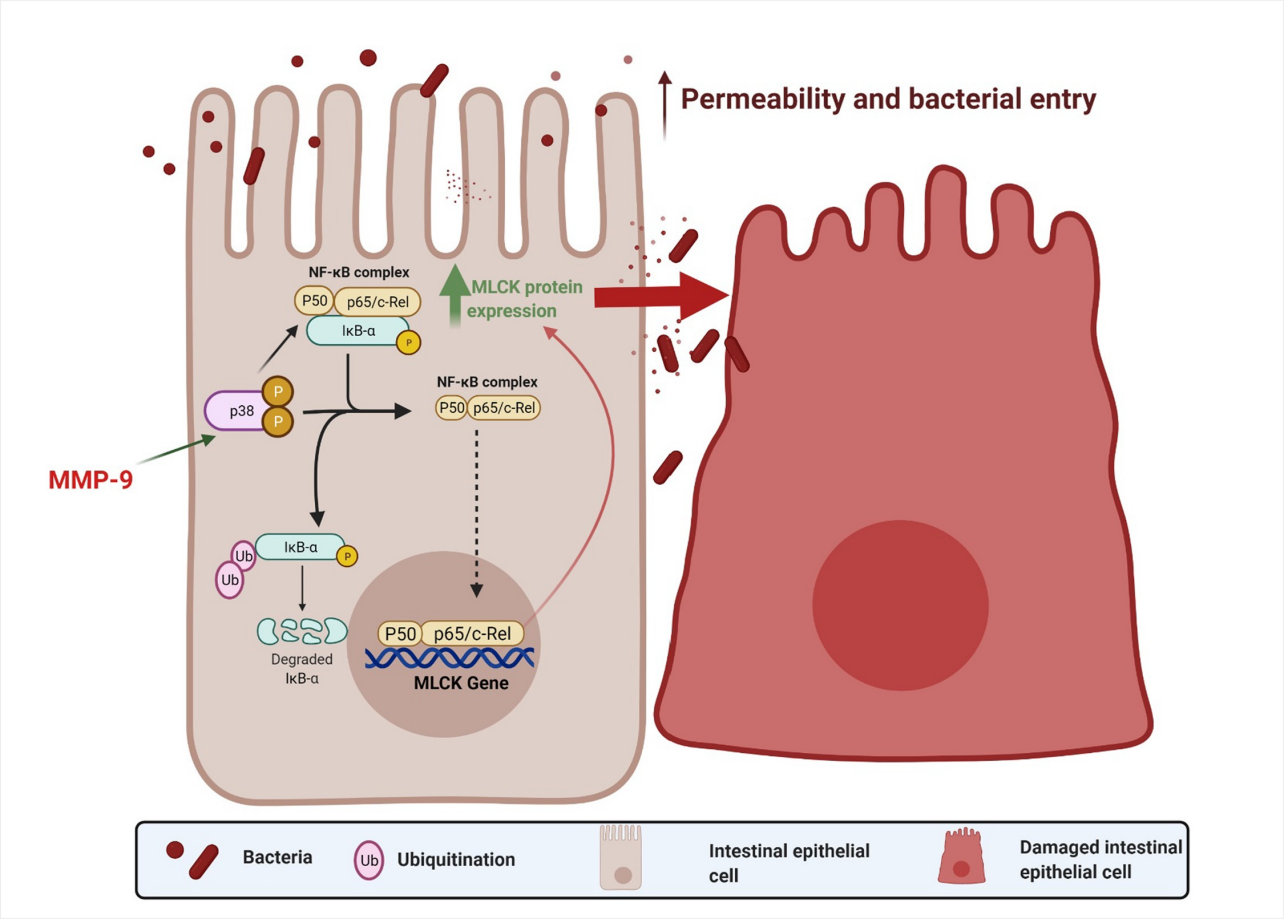
Schematic diagram of intracellular mechanism involved in MMP-9 regulation of Caco-2 TJ barrier function.

In conclusion, the current results demonstrated for the first time that NF-κB plays a key role in MMP-9 induced increase in MLCK expression and subsequently leads to an increase in intestinal epithelial TJ permeability in Caco-2 monolayers. An overall schematic diagram of the intracellular pathway examined in this study is shown in Fig 5.

## Supporting information

S1 Raw images (PDF)
