# Supplementary material for: Matrix Metalloproteinase-9 (MMP-9) induced disruption of intestinal epithelial tight junction barrier is mediated by NF-κB activation
Source: PLoS One. 2021 Apr 7;16(4):e0249544. doi: 10.1371/journal.pone.0249544 (PMC8026081; doi:10.1371/journal.pone.0249544)

ChemiDoc Gel Processing Ladder Fig. 2 A

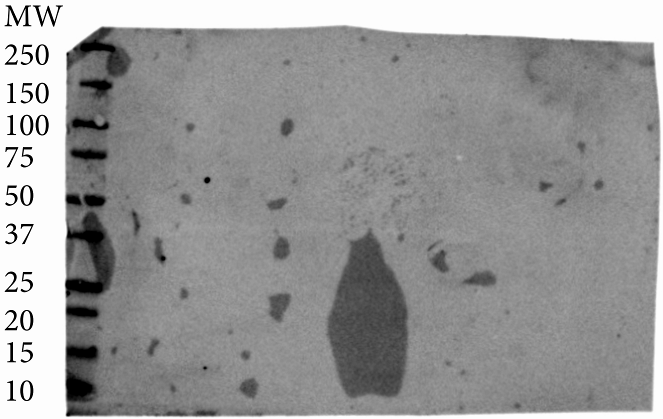

ChemiDoc Gel Processing Fig. 2 A-IκB-α

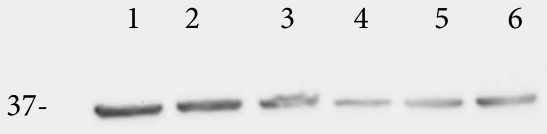

1: control Caco-2

2: MMP-9 (400 ng/ml)- 30 minutes

3: MMP-9 (400 ng/ml)- 1 hour

4: MMP-9 (400 ng/ml)- 2 hours

5: MMP-9 (400 ng/ml)- 4 hours 6: MMP-9 (400 ng/ml)- 6 hours

ChemiDoc Gel Processing Fig. 2 A-phospho-p65

1 2 3 4 5 6

65-

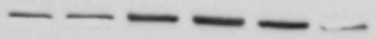

NF-κB p65

1: control Caco-2

2: MMP-9 (400 ng/ml)- 30 minutes

3: MMP-9 (400 ng/ml)- 1 hour

4: MMP-9 (400 ng/ml)- 2 hours

5: MMP-9 (400 ng/ml)- 4 hours

6: MMP-9 (400 ng/ml)- 6 hours

ChemiDoc Gel Processing Fig. 2 B Ladder

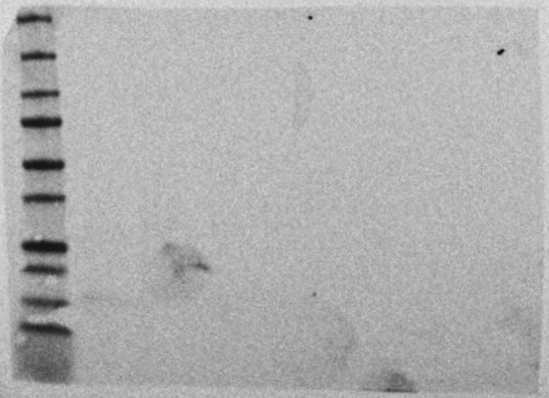

ChemiDoc Gel Processing Fig. 2 B NF-κB p65

65-

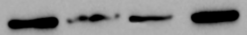

P65

1: control-cytoplasmic fraction
2: control-nuclear fraction
3: MMP-9 -cytoplasmic fraction
4: MMP-9 nuclear faction

ChemiDoc Gel Processing Fig. 2 E Ladder

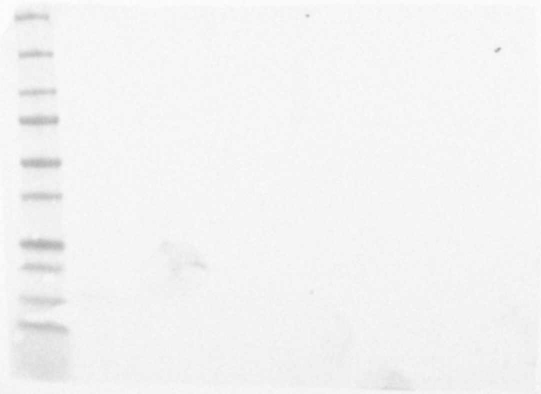

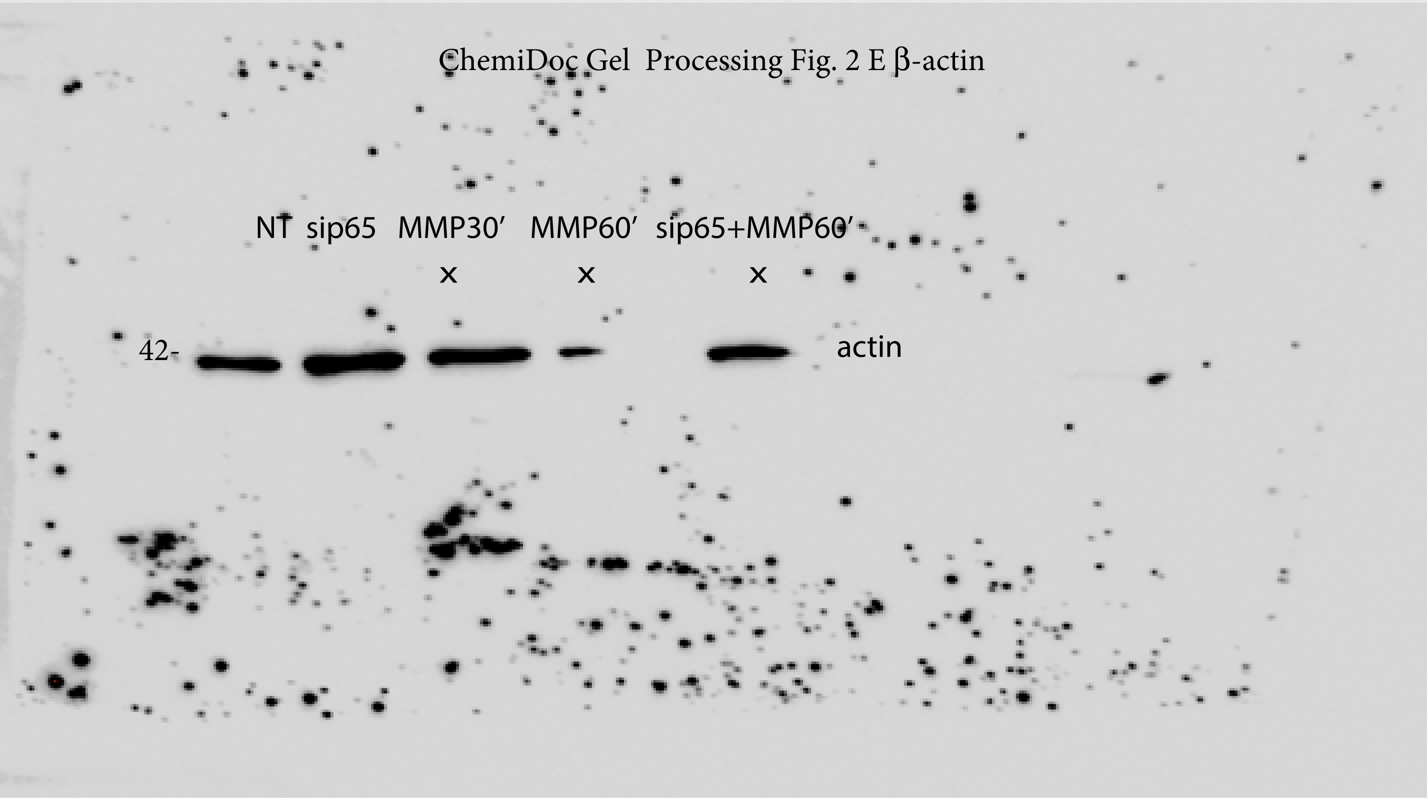

ChemiDoc Gel Processing Fig. 2 E β-actin

ChemiDoc Gel Processing Fig. 2 E NF-κB p65

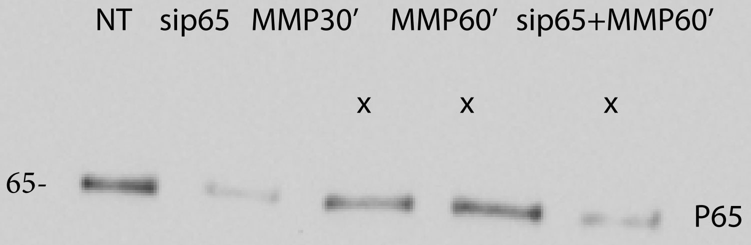

ChemiDoc Gel Processing Fig. 3 D Ladder

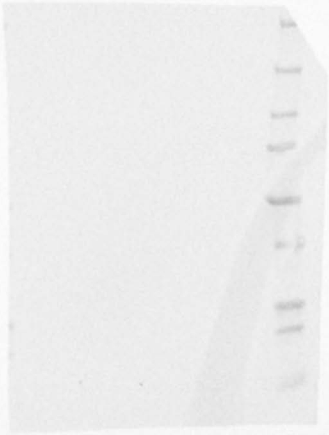

ChemiDoc Gel Processing Fig. 3 D MLCK

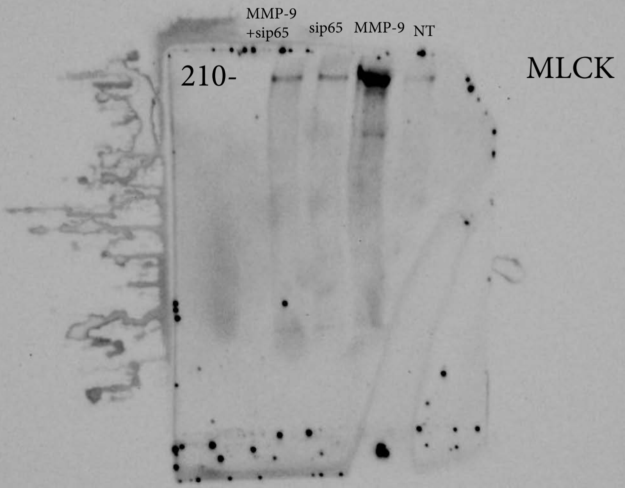

ChemiDoc Gel Processing Fig. 3 D β-actin

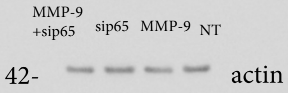

ChemiDoc Gel Processing Fig. 3 E β-actin

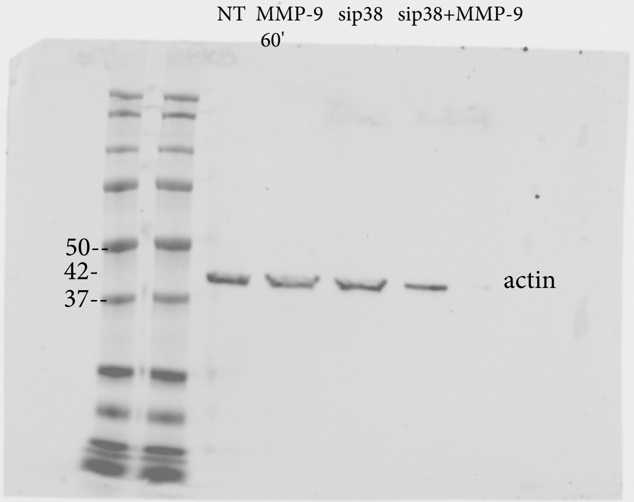

ChemiDoc Gel Processing Fig. 3 E p38 kinase

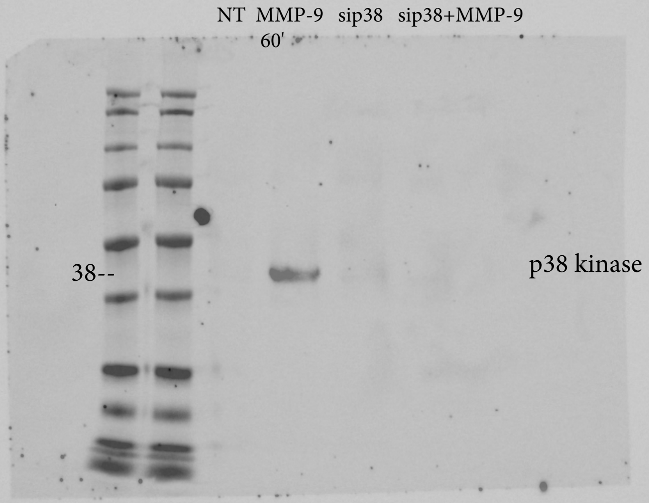

ChemiDoc Gel Processing Fig. 3 E Ladder

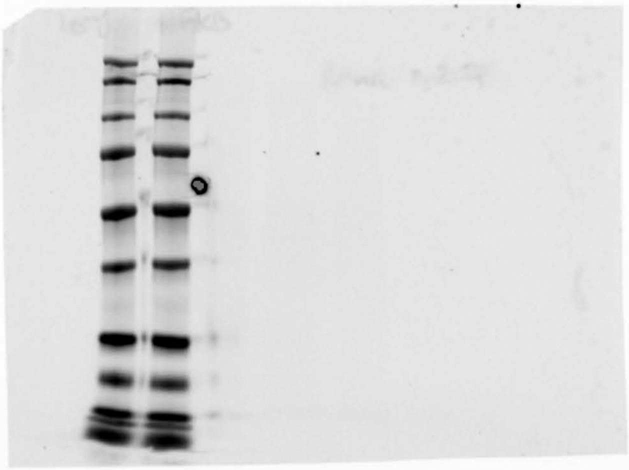

ChemiDoc Gel Processing Fig. 3 E β-actin

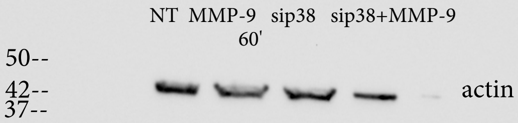

ChemiDoc Gel Processing Fig. 3 E p38 kinase

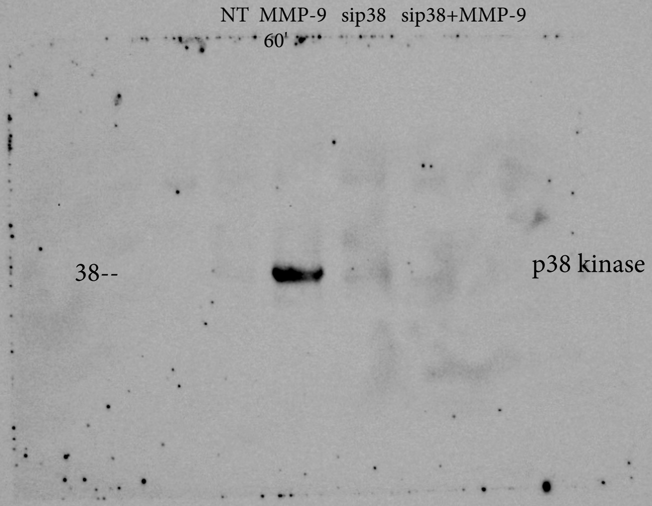

Supplement: S1 Raw images — (PDF) [file pone.0249544.s001.pdf]
